# Can cholesterol and its products serve as biomarkers for *ojas*?

**DOI:** 10.1016/j.jaim.2025.101240

**Published:** 2025-11-24

**Authors:** Venil N. Sumantran, Pratibha P. Nair

**Affiliations:** a Dr. A.P.J. Abdul Kalam Centre for Excellence in Innovation & Entrepreneurship., Dr. M.G.R. Educational and Research Institute, Maduravoyal, Chennai, 600095, India; b VPSV Ayurveda College, Kottakkal, Malappuram, Kerala, 676501, India

**Keywords:** Atherosclerosis, HDL-C, LDL-C, Hypocholesterolemia, Vitamin D

## Abstract

*Ojas* is essential for human heath and vitality. In 1995, Walton et al. proposed an equivalence between ojas and cholesterol, and linked specific dhatus with steroid hormones derived from cholesterol. This paper examines five links between cholesterol and ojas. First, properties of ojas suggest a complex, mobile or immobile lipid. Only cholesterol has these properties. Insoluble, unesterified cholesterol maintains cell membranes. Cholesterol synthesized by the liver is converted into soluble cholesteryl lipoprotein esters (LDL-C and HDL-C), which circulate like apara ojas. Interestingly, cholesteryl-esters and apara ojas respond to diet and drugs. Second, eight biomolecules (five steroid hormones, bile acids, vitamin D, and myelin), which can only be synthesized from cholesterol, function at twelve physiological sites of ojas activity. Third, cholesterol and its products explain key functions of ojas. Thus, cholesterol controls reproduction, fertilization (*shukra*), and has a morphogenetic role in foetal development (*garbhasara*). Cholesterol and its products control stress responses, brain functions, and the musculoskeleton (*bala*). Cholesterol immunometabolism and vitamin D regulate immunity (*vyadhi kshamatva*). Fourth, oxidized LDL-C contributes to plaque formation in atherosclerosis, which is the leading cause of global deaths. Conversely, hypocholesterolemia and depleted ojas (*ojo kshaya*) significantly increase risk of mortality. Fifth, long term studies suggest that HDL-C can be a surrogate marker of healthy *apara ojas*. These facts indirectly prove that cholesterol homeostasis and robust ojas are absolute requirements for health and survival. We explain how these five links provide correlative, potentially causal, and clinical evidence for our hypothesis that cholesterol and products of cholesterol, are candidate biomarkers for ojas.

## 1 Introduction

*Ojas* represents optimum functioning of bodily tissues and defences against internal and external factors that disrupt our innate balance. Biological functions of *ojas* vary across ayurvedic texts, where it is considered the basis for life (*pranaayatana*), circulating nutrients (*shareera rasa sneha*), normal state of *kapha dosha*, essence of *dhatus* (*dhathu sara*), byproduct of metabolism (*dhathu mala*), supportive to tissues (*upadhathu*), and the basis of immunity (*vyadhi kshamatva*). *Ojas* is non-reactive, inherently stable (*somaatmaka*), and can be enhanced by dietary interventions [1], Sootra Sthana/Chapter 15/Verse21]. Answering provocative questions (PQs) (https://provocativequestions.nci.nih.gov/) revealed that vagus nerve activity could represent a biomarker for some functions of vata dosha [2]. Here, we raise 10 PQs for identifying a candidate biomarker for *ojas*. Answers to the 10 PQs uphold an earlier hypothesis which proposed an ‘equivalence between *ojas* and cholesterol’ [3].

### 1.1 PQ-1: what evidence links *KaphaPrakriti*, *ojas,* and cholesterol?

*Kapha Prakriti* and Cholesterol: *Kapha Prakriti* demonstrates bahu ojas [4, Shareera Sthana/Chapter 3/Verse 98] and optimal levels of ojas (oja vruddi) causes *tushti*/*santosha* (pleasure), *pushti* (good nourishment), *sthoulya* (obesity), *meda* (increased BMI), and enhanced *bala* (saamarthya/shakti utkarsha) [4, Ayurveda *Rasayana vyakhya* on Sootra Sthana/Chapter 11/Verse 41]. Interestingly, genomic analysis of healthy individuals found an increase in total cholesterol and LDL-cholesteryl esters in males with kapha prakriti compared to males with *vata* or *pita prakriti* [5]. This positive correlation between ojas, cholesterol, and *kapha prakriti*, supports a proposed ‘equivalence between cholesterol and *ojas*’ published in 1995. This equivalence was based on physico-chemical properties and physiological functions shared by *ojas*, cholesterol, and products of cholesterol [3].

### 1.2 PQ-2: Why cholesterol?

Can't other complex lipids represent *ojas*?

Properties of complex lipids and *ojas*:

Phospholipids, sphingolipids, glycolipids, and cholesterol are complex lipids in humans. Phospholipids comprise the ‘lipid bilayer’ in eucaryotic cell membranes. Some phospholipids and sphingolipids control cell growth, death, and differentiation. Glycolipids and sphingolipids regulate cell adhesion and communication. Ojas is shleshma, and its properties (*gunas*) include ‘soft’ (*mrdu*), ‘smooth’ (*slakshna*), ‘unctuousness’ (*snigdha*), ‘heaviness’ (*guru*), ‘compactness/stability’ (*sthira*), ‘heat sensitivity’ (*sheeta*), and ‘mobile/flowing’ (*sara*), [6,7]. These *gunas* suggest that *ojas* is a complex, waxy lipid with immobile and mobile forms. Cholesterol is the only complex lipid which exists in such forms and performs functions of *ojas*.

Cellular and Circulating Forms of Cholesterol and *Ojas*: ‘*Ojas* is present in every tissue of the body in dormant form and is manifested as supreme quality after its proper metabolism’ [6]. Similarly, dietary cholesterol is absorbed as the culmination of lipid digestion by the intestines. The liver also synthesizes significant amounts of cholesterol daily [8]. *Ojas* is the sara/essence of all dhatus. It is located in hrdaya, circulates with rasa, and performs jeevana and tarpana of the entire body [7]. Thus, *ojas* and cholesterol are within cells/organs and in circulation, and cholesterol has different forms at these sites. Insoluble, unesterified cholesterol (UC) maintains structure and selective permeability of all human cell membranes. Importantly, UC within specialized ‘lipid rafts’ surrounds membrane receptors and regulates cellular signalling [9]. Dietary and liver cholesterol are converted into soluble, neutral, cholesteryl-low density lipoprotein esters (LDL-C) which enter all organs via blood [8]. Excess cholesterol effluxed (exported) from cells circulates as cholesteryl-high density lipoprotein esters (HDL-C), and returns to the liver by ‘reverse transport’ activity of HDL-C [9].

Therefore, cellular and circulating cholesterol exist as UC and cholesteryl-esters respectively. Circulating cholesteryl-esters may represent circulating *ojas* as *rasatmaka* *ojas* or *apara ojas* [10], Sootra Sthana/Chapter17/Verse 117]. Notably, cholesteryl-esters and *apara ojas* respond to diet and drugs [4, Sarvanga Sundari vyakhya on Sootra Sthana/Chapter 5/Verse 20; 4, Sootra Sthana/Chapter 11/Verse 39; 10, Sootra Sthana/Chapter 27/Verse 214; 4, Sootra Sthana/Chapter 6/Verse 15; 4, Sootra Sthana/Chapter 10/Verse 6–9; 4, Sootra Sthana/Chapter 15/Verse 8]. Like *ojas*, cellular and circulating cholesterol have different gunas. Snigdha and sthira describe cellular cholesterol (UC), whereas *slakshna* and *sara* describe circulating cholesteryl-esters (LDL-C and HDL-C). Notably, *guru, mrdu*, and *sheeta* are gunas shared by *ojas*, UC, and circulating cholesterol [6,7]. Cholesterol homeostasis requires coordinated regulation of cellular cholesterol (UC), circulating cholesteryl-esters, and hepatic cholesterol. Optimal balance of these different forms of cholesterol may represent *oja vruddhi*, which is protective [4, Ayurveda *Rasayana vyakhya* on Sootra Sthana/Chapter 11/Verse 41].

### 1.3 PQ-3: Atherosclerosis is caused by abnormal cholesteryl-esters. Does this weaken the hypothesis linking cholesterol with *ojas*?

Atherosclerosis Proves the Importance of Cholesterol Homeostasis: The prevailing view is that atherosclerosis is caused by elevated LDL-C (hypercholesterolemia). However, data from 68,000 elders found that those with the highest LDL-C levels lived longer than those on statins [11]. Data on 140,000 patients admitted to hospital with acute myocardial infarction, found that they had low LDL-C levels [11]. Therefore, there is controversy regarding a causal role for high LDL-C levels in atherosclerosis. However, there is agreement that abnormal localization and activity of oxidized LDL-C [9,12] contributes to formation of ‘foam cells’ which form plaques in blood vessels of atherosclerosis patients. In this context, we propose that *oja visramsa* refers to *sthaan chyuti*/displacement from normal sites, and may correspond to excessive displacement of LDL-C from liver into circulating blood. *Oja vyapat* refers to altered characteristics (*gunas*) and functions (*karma*) inflicted by *doshas*, and may correspond to macrophages which accumulate oxidized LDL-C (‘foam cells’). Importantly, foam cells and inflammatory factors produce atherogenic lesions in arteries [9,12].

Therefore, mechanisms by which displaced LDL-C and oxidized LDL C cause atherosclerosis can explain stage wise pathology of *ojas* (*ojas vikara*), namely *oja visramsa* and *oja vyapat*, in atherosclerosis. Atherosclerosis increases risk of mortality because patients have concomitant vascular and immune changes which damage vital organs, bone marrow, brain, and gastrointestinal tract [13]. This widespread multi-organ damage explains why atherosclerosis is the leading cause of global deaths, and provides indirect clinical evidence for the fundamental importance of normal metabolism and homeostasis of cholesterol. Similarly, robust *ojas* is the primary determinant of good health [6,7]. These facts strengthen (not weaken), the hypothesis that cholesterol represents *ojas* [3]. Mechanisms by which cholesterol and *ojas* sustain life are explained below.

### 1.4 PQ-4: Eight essential biomolecules can only be synthesized from cholesterol. Do these 8 products of cholesterol perform functions of *ojas*?

**Fig. 1:**
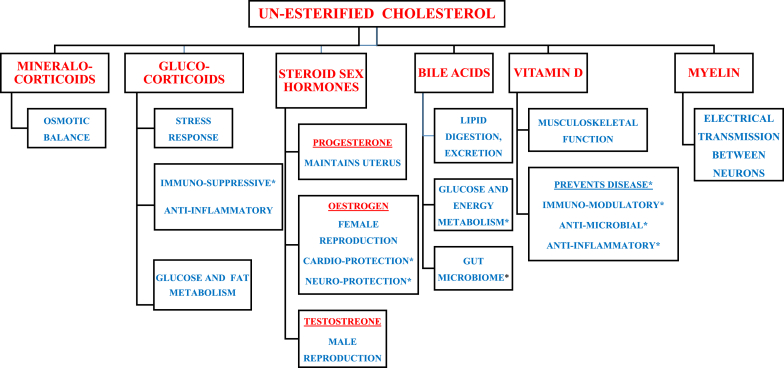
Products of unesterified cholesterol (UC): Eight essential biomolecules are produced from UC. Primary and secondary (∗) functions of each Biomolecule is Shown.

Biomolecules derived from cholesterol: Certain organs synthesize specialized biomolecules from cholesterol when required. Thus, five steroid hormones (glucocorticoids, mineralocorticoids, and three sex hormones) derived from cholesterol have important primary and secondary homeostatic functions in osmotic balance, energy metabolism, stress responses, immunity, and reproduction (Fig. 1). Cholesterol is also the sole precursor of bile acids, vitamin D, and myelin [9] (Fig. 1). Interestingly, excess cholesterol is not degraded, but forms bile acids which promote lipid digestion and absorption of fat-soluble vitamins [9]. Bile acids were thought to regulate lipid metabolism, and abnormal bile acid secretion was linked to certain hepatic and cholestatic diseases. However, interactions between bile acids and the farnesoid X receptor (FXR), and interactions between bile acids and the gut microbiome were discovered [14]. These interactions enable bile acids to also regulate glucose and energy metabolism. Accordingly, abnormalities in bile acid interactions and bile acid transport, aggravate diabetes mellitus, obsesity, and gut inflammation. Therefore, drugs which sequester bile acids improve glycemic control in diabetics. Similarly, bariatric surgery improves bile acid metabolism and homeostasis, which then improves glycemic control in obese patients [14].

Notably, *madhumeha* is a type of *prameha* with direct causal involvement of *ojas*. *Ojo vyapat* is causally linked with *madhumeha*, making it *asadhya* (poor prognosis) [10, Chikitsa Sthana/Chapter 6/Verse 57]. Vitamin D is required for bone development and immunomodulation [15], and myelin facilitates neural signalling (Fig. 1). To summarize, eight biomolecules synthesized from cholesterol (cholesterol's products), perform vital cellular, metabolic, and physiological processes which cannot be performed by other biomolecules (Fig. 1). Walton et al. matched cholesterol's products with specific *dhatus*. For example, *rasa*, *medas*, and *asthi dhatus* were correlated with bile acids, glucocorticoids, and vitamin D3 respectively [3]. Hemadri cross linking Susrutha's concept on *ojas* mentions ‘*Dhathunam tejasi rase tatha jeeva shonite*’, indicating circulating derivatives of *poshaka rasa* (nutrients) for *dhathu poshana* (nutrition for forming successive dhathus). Thus, cholesterol's products may correlate with Dhatu tejo rupi ojas which nourishes and maintains unique and specific properties of each *dhatu* [4, Ayurveda *Rasayana vyakhya* on Sootra Sthana/Chapter 11/Verse 37–39].

### 1.5 PQ-5: Do cholesterol and its products and *ojas*, function at the same physiological sites?

Cholesterol and its products function at 12 Sites of *oja*s activity: If *ojas* and cholesterol have similar functions and activities, one expects that both entities, their byproducts, and waste, would be localized to similar sites. PQ-5 explains how *ojas*, cholesterol, and its products, are active in 12 sites as described by Acharya Bhela [16], Sootra Sthana/Chapter 12/Verse 7]. These12 sites of *ojas* activity include: 1. *kapha* (mucous, synovial fluid, cell membrane, lubricants and nutrients), 2. *pitta* (metabolism/enzymes) 3. *rakta* (circulating blood), 4. *medas* (adipose tissue). 5. *mamsa* (muscle), 6. *asthi-majja* (bone-marrow), 7. *shukra mala* after *shukra paaka* 8. *para ojas*, *shukra-shukla* (gametes and seminal fluid). 9. *garbhasara* (foetal life and birth) 10. *mutra* (urine), 11. *pureesha* (stools) 12. *sweda* (sweat). Links between cholesterol and *kapha*, *rakta*, and *medas* were explained (PQ-1, 2). Cholesterol metabolism is part of lipid metabolism which correlates with *pitta sthanas*, *jatharagni* and *dhathu agni* [10, Chikitsa Sthana/Chapter 15/Verse 3-9-11]. Although cholesterol is absent in urine, steroid hormones are sometimes present in urine (*mutra*), as is *ojas.* Excess bile acids and ojas are excreted in faeces (pureesha). Cholesterol and *ojas* are found in sweat (sweda).

Thus, cholesterol (and/or its products) function in 4 sites of *ojas* activity and are excreted into 3 waste materials where *ojas* is found. The remaining 5 sites of cholesterol and *ojas* activity are discussed in PQ-6 and PQ-7. It is remarkable that *ojas*, cholesterol, or certain products of cholesterol, are active and localized in the same 12 sites. This similarity would arise if these entities are processed via common metabolic pathways and are transported similarly though the same organs. Thus, PQ-5 strongly supports the hypothesis linking *ojas* to cholesterol and its products. PQ-5 also highlights that cholesterol and its products have different, but equally important links to *ojas*.

### 1.6 PQ-6: Reproduction (*shukra*) and birth (*garbhasara*) require ojas. Does cholesterol regulate *shukra* and *garbhasara*?

Walton et al. highlighted similarities between *shukra dhatu* and sex hormones derived from cholesterol [3]. These sex hormones regulate functions of reproductive organs (Fig. 1). However, cholesterol has additional functions in reproduction.

Cholesterol controls fertilization and foetal development:

Cholesterol content of seminal fluid positively correlates with sperm count [18]. Cholesterol also maintains rigidity of sperm. During ‘capacitation’, timely removal of a sperm's cholesterol triggers molecular events which make sperm flexible, motile, and ready to fertilize an ovum [19]. Therefore, cholesterol directly controls sperm count and sperm activity. Notably, *ojas* is a derivative of *shukra* [10, Sootra Sthana/Chapter 30/Verse 9] and *para ojas* refers to *mala* (byproduct), when *shukra* (paternal gamete) undergoes *paka* (metabolic/structural changes) in the presence of *artava* (maternal gamete) during fertilization [10], Sootra Sthana/Chapter 30/Verse 9; [17], Sootra Sthana/Chapter 19/Verse 27 [4]; Shareera Sthana/Chapter 3/Verse 63]. Thus, *shukra paka* may correspond to the sperm activation mechanism (capacitation), and *para ojas* (*shukra mala rupi para ojas*) may represent cholesterol released during capacitation [1], Nibandha Samgraha Vyakhya on Sootra Sthana/Chapter 15/Verse 21–22; and 4, Ayurveda *Rasayana Vyakhya* on Sootra Sthana/Chapter 11/Verse 37–38]. After fertilization of the ovum by sperm, the embryo/foetus develops with placental cholesterol, sex hormones, vitamin D, myelin, and other nutrients [8,20]. Since the embryo grows and develops rapidly, there is high demand for placental and foetal cholesterol during prenatal growth. Importance of this cholesterol is proven by the fact that only 1 in 20,000 human embryos with inborn errors in cholesterol biosynthesis are born. These rare infants suffer from Smith-Lemli-Opitz syndrome, which is characterized by several facial and cardiac congenital abnormalities, and mental retardation, due to cholesterol deficiency [9].

Therefore, placental cholesterol has a direct role as a developmental ‘morphogen’. Placental cholesterol also binds and activates the Sonic Hedgehog (Shh) protein. Active Shh regulates development of foetal spinal neurons and also activates specific transcription factors which regulate expression of foetal genes that control development of the foetal head, limbs, lungs, heart, and urogenital systems [20]. In summary, cholesterol is a developmental ‘morphogen’ which directly controls normal foetal growth and development. Cholesterol also indirectly regulates foetal development by controlling activity of Shh proteins. These morphogenetic properties of cholesterol in foetal development could represent garbhasara. *Apara ojas* from the mother (*Rasatmaka* *ojas*) is transported to the foetus, and may represent maternal/placental cholesterol which sustains foetal development until the foetus synthesizes cholesterol de-novo [9,20] *Jeeva shonita rupi para ojas* produced during or after fertilization sustains foetal development [4, Ayurveda *Rasayana Vyakhya* on Sootra Sthana/Chapter 11/Verse 37–38], and may correspond to foetal cholesterol synthesized and utilized by the foetus [20].

To summarize, *ojas* is derived from *shukra* [10, Sootra Sthana/Chapter 30/Verse 9], and *jeeva shonita rupi para ojas* sustains foetal development [4, Ayurveda *Rasayana Vyakhya* on Sootra Sthana/Chapter 11/Verse 37–38].

Similarly, cholesterol plays an essential biochemical role in sperm activation and fertilization, and has direct and indirect morphogenetic roles in normal foetal development [9,[18], [19], [20]]. Of all the complex lipids mentioned in PQ-2, only cholesterol has specialized roles in sperm function, embryonic development, and morphogenesis.

### 1.7 PQ-7: *Bala* (musculoskeleton and Brain functions) requires *ojas*. Do cholesterol and its products regulate *Bala*?

Musculoskeletal and Brain Functions: *Ojas* provides *bala*, and joint dysfunction and weakness are manifestations of displaced *ojas* (*oja visramsa*). Interestingly, cholesterol is essential for muscle cell proliferation and movements of zebra fish embryos [21]. Bones are calcified by vitamin D and myelin regulates activity of nerves which control joint movement. *Bala* maintains cognition and mental health [10, Shareera Sthana/Chapter 6/Verse 13]. The brain is enriched with cholesterol and myelin, and Walton et al. correlated *sattva dhatu* (consciousness and mental faculties), with specific neuro-steroids derived from cholesterol [3]. Notably, dysregulated cholesterol homeostasis in the brain significantly correlates with onset of stroke, schizophrenia, depression, and neuro-degenerative disorders [22]. Similarly, vitiated ojas (oja vyapath) causes senility, fear, anger, depression, and grief [7]. Depleted *ojas* (*oja kshaya*) causes incoherence and unconsciousness [6]. In contrast, *ojo vruddhi* causes *tushti* (*santosha*, *chintaadi rahita*) [4, Sootra Sthana/Chapter 11/Verse 4]. Thus, optimal functioning of ojas is associated with normal cognition and pleasure.

To summarize, *ojas* causes *bala*.

In fact, *ojas* and *bala* are considered synonymous [4, Sootra Sthana/Chapter 11/Verse 41]. Similarly, cholesterol, vitamin D, and myelin have causal roles in normal growth, development, and functioning of the musculo-skeleton and brain (Fig. 1) [9].

### 1.8 PQ-8: *Vyadhi kshamatva* (immunity) is the foremost attribute of *ojas*. Do cholesterol and its products Regulate immunity?

*Ojas* acts across different bodily systems (*sakala shareera vyapi*) and is associated with all *dhathus* (*rasaadinam shukrantanam yat param teja, tat balam*) [1, Sootra Sthana/Chapter 15/Verse 23]. Ojas also causes deha bala (*samarthya/shakti utkarsha*) or immunity (*vyadhi kshamatva*), since it provides substances in rasa and rakta which protect dhatus from decay and degeneration, and prevent disease [7].

Cholesterol and its products preserve health and prevent disease:

Cholesterol preserves all organs by maintaining structure and function of cell membranes. Steroid hormones derived from cholesterol regulate metabolic, homeostatic, and reproductive functions, and oestrogen also has protective functions [23]. Bile acids have multiple functions. Cholesterol, vitamin D, and myelin preserve health by performing essential functions for *shukra*, *garbhasara*, and *bala* (PQs 6,7) (Fig. 1). All cells require cholesterol, but active immune cells accumulate cholesterol by ‘cholesterol immune-metabolism’ wherein cholesterol synthesis increases and cholesterol efflux decreases [12]. This accumulated cholesterol promotes proliferation and activation of T-cells, monocytes, and neutrophils during infections. Cholesterol also promotes haematopoiesis and myelopoiesis by stimulating proliferation and mobilization of hematopoietic stem cells within bone marrow [12], and this may represent *ojas* activity in *asthi-majja* [16, Sootra Sthana/Chapter 12/Verse 7]. However, prolonged accumulation of cholesterol within these immune cells causes hypersensitivity, chronic inflammation, and formation of foam cells [12] (PQ3). Therefore, controlled ‘cholesterol immune-metabolism’ prevents disease by regulating activity of immune cells and hematopoietic stem cells [12]. Glucocorticoids and Vitamin D also prevent disease (Fig. 1). Glucocorticoids are anti-inflammatory and immunosuppressive. Vitamin D prevents infections, autoimmune diseases, certain cancers, metabolic syndrome, mood disorders, and infertility [9,15]. Notably, meta-analyses show that vitamin D deficiency significantly correlates with increased risk of mortality [15]. Therefore, like *ojas*, cholesterol and its products are essential for health preservation and disease prevention (*vyadhi kshamatva*).

### 1.9 PQ-9: Cholesterol maybe required for *vyadhi kshamatva*. But is there clinical evidence for protective functions of cholesterol?

Hypocholesterolemia increases risk of Mortality and HDL-C Is Protective: Levels of total cholesterol and LDL-C significantly decrease during serious illness and increase in patients who recover [8]. It is unclear whether this hypocholesterolemia is a cause or consequence of critical illness. However, persistent hypocholesterolemia is a risk factor for morbidity and mortality in sepsis and adrenal failure, and cholesterol should be monitored in patients with severe trauma [8]. Therefore, hypocholesterolemia and depleted *ojas* (*oja kshaya*) are significantly correlated with disease progression and mortality [1, Sootra Sthana/Chapter 15/Verse 24]. Notably, increased levels and activity of HDL (reverse cholesterol transport), are cardioprotective [12] and neuro protective [22,24].

### 1.10 PQ-10: Regulation of metabolism of cholesterol and its products is complex. So, how can cholesterol be a reliable biomarker of *ojas*?

HDL Cholesteryl Esters can be reliable biomarkers of *Apara Ojas*: Biomarkers must be measurable, and cholesterol within cell membranes cannot be easily measured. Cholesterol's products are also unsuitable biomarkers of ojas because their levels depend on factors besides cholesterol. For example, synthesis of sex hormones and myelin depends on age, gender, and diet. Synthesis and levels of vitamin D3 depend on exposure to sunlight, diet, and efficiency of fat absorption. A surrogate endpoint is 'a biomarker intended to substitute for a clinical endpoint', a surrogate endpoint does not measure the clinical benefit of primary interest in and of itself, but rather is expected to predict that clinical benefit’. These 2 definitions imply that surrogate markers give an indirect but accurate estimation of the presence/progression of a clinical condition. In PQ 3 we cite literature that high LDL-C levels may not have a causal link with atherosclerosis. However, oxidized LDL-C contributes to formation of atherogenic plaques [9,12], and could be a potential surrogate marker for abnormal *apara ojas* and atherosclerosis.At present, this is not realistic since methods for measuring oxidized LDL-C are not suitable for clinical use.

Recent evidence suggests that HDL-C activity (reverse cholesterol transport), is a more reliable predictor of cardiovascular functions of HDL-C, than HDL-C levels [24]. However, some long-term studies find protective effects of high levels of HDL-C. For e.g., a Scandinavian study tracked cardiovascular risk factors for 40 years. Using ultrasound to measure plaque in carotid arteries of 20,000 people, they found that high levels of HDL-C were protective [25]. One Japanese study tracked 1299 people for mild cognitive impairment (MCI) and dementia for 19 years. Results showed that high HDL-C levels in midlife were significantly correlated with later onset of MCI and dementia [26]. Other studies also report that increased HDL-C levels protect against Alzheimer's disease [22,24]. A Biobank in the UK analyzed 118,021 individuals and found that increased levels of small HDL-C particles significantly correlated with decreased risk of stroke, as confirmed by MRI scans. In contrast, large HDL-C particles were associated with the opposite effect [27]. Therefore, although the levels of cholesterol, LDL-C and HDL-C are influenced by age, gender, and diet, these careful long-term studies strongly suggest that HDL-C can be a surrogate marker of cardio-protection and neuro-protection for some diseases. Accordingly, we hypothesize that normal HDL-C levels could be a surrogate marker of healthy Apara Ojas.

Table 1 gives a comprehensive summary of the 10 PQs with answers. It gives pertinent biochemical and physiological links between cholesterol, products of cholesterol, and *Ojas*.

**Table 1:** List of provocative questions [PQs] and their answers.

	Provocative Questions [PQs]	Answers To PQs
**PQ-1:**	What Evidence Links *Kapha Prakriti*, *Ojas*, and Cholesterol?	*Kapha Prakriti* demonstrates *bahu ojas.* Genomic Analysis links Males of K*apha Prakriti* with increased levels of total Cholesterol and LDL-Cholesteryl esters.
**PQ-2:**	Why Cholesterol ? Can't Other Complex Lipids Represent *Ojas*?	*Gunas* of *Ojas* describe a complex Lipid with Immobile [s*nigdha* and *sthira*] and Mobile [*slakshna* and *sara*] forms. Cholesterol is the only complex lipid with immobile and circulating forms [LDL-C, HDL-C]. Only Cholesterol and its Products perform functions of *Ojas*.
**PQ-3:**	Atherosclerosis is Caused by Abnormal Cholesteryl-Esters. Does this Weaken the Hypothesis Linking Cholesterol and *Ojas*?	Atherosclerosis Is The Leading Cause of Global Deaths. This Strengthens Our Hypothesis that Normal Metabolism and Homeostasis of Cholesterol Are Essential for Good Health.
**PQ-4:**	Eight Biomolecules Are Only Synthesized from Cholesterol. Do these Products of Cholesterol Perform Functions of *Ojas*?	Eight Products of Cholesterol: Five Steroid Hormones Control Homeostasis, Immunity, and Reproduction. Bile Acids Regulate Energy Metabolism And Vitamin D controls Bone Development and Immunity. Myelin controls Nerve Signalling (Fig. 1).
**PQ-5:**	Do *Ojas,* Cholesterol, And its Products Function at the Same Physiological Sites?	*Ojas,* Cholesterol and its Products, Are Active and Localized at the Same 12 sites. Therefore, O*jas,* Cholesterol, and its Products, could be Metabolized and Transported Similarly Through Our Organs.
**PQ-6:**	Reproduction (S*hukra*) and Birth (G*arbhasara*) Require *Ojas*. Does Cholesterol Regulate *Shukra* and *Garbhasara?*	Capacitation may represent *shukra paka*. Maternal A*para Ojas/Rasatamaka Ojas* may represent Placental Cholesterol. Foetal Cholesterol may represent *Jeeva Shonita Rupi Para Ojas.* Cholesterol is a ‘Morphogen’ During Foetal Development [*Garbhasara*].
**PQ-7:**	*Bala* (Musculoskeleton and Brain Functions) Requires *Ojas*. Do Cholesterol and its Products regulate *Bala*?	*Ojas* causes *Bala.* Similarly, Cholesterol, Vitamin D, and Myelin have Causal Roles in Normal Growth and Functions of the Musculoskeleton and Brain (Fig. 1)
**PQ-8:**	*Vyadhi kshamatva* (immunity) is the foremost attribute of *Ojas.* Do Cholesterol and its Products Regulate Immunity*?*	‘Cholesterol Immunometabolism’ Regulates Immune Cell Activity. O*jas*, Cholesterol and its Products Preserve Health And Prevent Disease (*vyadhi kshamatva*).
**PQ-9:**	Cholesterol Maybe Required for *Vyadhi kshamatva*. But, is there Clinical Evidence for Protective Functions of Cholesterol?	Hypocholesterolemia and Depleted O*jas* (O*ja Kshaya*) Significantly Correlate with Disease Progression and Mortality. Increased Levels and Activity of HDL-C are Cardioprotective and Neuroprotective.
**PQ 10:**	Regulation of Metabolism of Cholesterol and its Products is complex. So, how can Cholesterol be a Reliable Biomarker of *Ojas*?	Cholesterol And its Products are Not Reliable Biomarkers of *Ojas.* Long Term Studies suggest that HDL-C Can Be a Surrogate Marker of Healthy *Apara Ojas*.

### 1.11 Summary of evidence on cholesterol and ojas

**Fig. 2:**
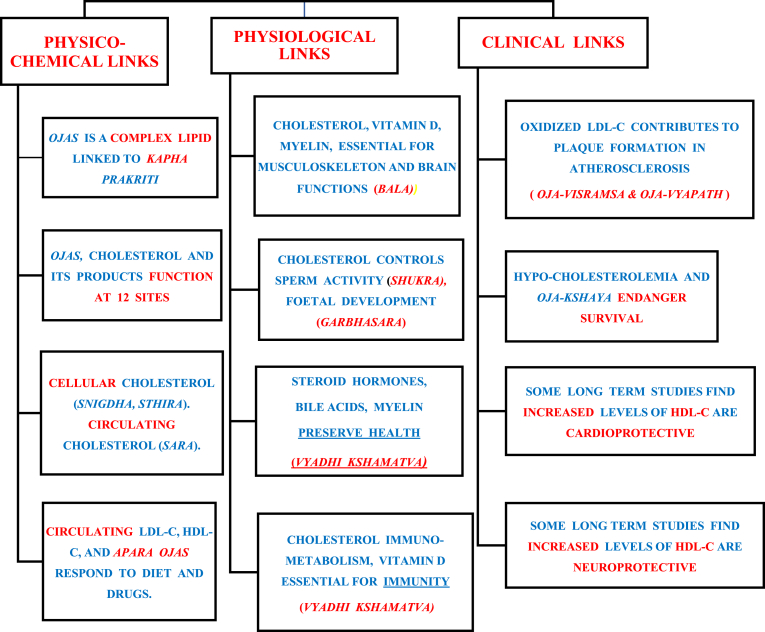
Links between Ojas and Cholesterol and its Products: Physico-chemical links give correlative evidence. Physiological links give potentially Causal and Clinical evidence.

Walton et al. [3] and our study propose that a complex lipid represents ojas. If accepted, then, cholesterol is the best candidate because of comprehensive evidence linking ojas to cholesterol and its products (Fig. 1, Fig. 2). Physico-chemical links between cholesterol and ojas are correlative (Fig. 2). Physiological processes required for *bala*, *shukra*, *garbhasara*, *vyadhi kshamatva*, are similar to processes which can only be perfomed by cholesterol and its products (Fig. 2). Regarding *bala*, we explain how cholesterol, vitamin D, and myelin control normal functions of the musculo-skeleton and brain, and note that similar abnormalities of bala arise due to impaired ojas and dysregulated cholesterol homeostasis (PQ 7) (Fig. 1), [9]. Similarly, there are physiological links between *shukra* and cholesterol, maternal *apara* *ojas/rasatamaka ojas* and placental cholesterol, *jeeva shonita rupi para ojas* and foetal cholesterol. Regarding *vyadhi kshamatva*, the importance of cholesterol immune metabolism in immunity is discussed, and clinical evidence for protective roles of oestrogen, vitamin D, and HDL-C is cited (PQs 8,9).

Therefore, there is biochemical and physiological evidence for potentially causal links between cholesterol, products of cholesterol, and each major function of *Ojas* [musculoskeletal health, reproduction, and immunity. Functions of genes/proteins are established by inhibiting the gene or protein in a defined cell population, and measuring the resulting changes. Since these cellular changes arise from loss of function of the gene/protein, one can infer functions of said gene/protein. Similarly, functions of *ojas* and cholesterol can be clarified by comparing effects of depleting cholesterol and *ojas*. Notably, three important potentially causal links between cholesterol and *ojas* emerge when data on hypo-cholesterolemia is compared with *ojo kshaya*. First, cholesterol deficiency inhibits cholesterol immune metabolism, immunity, and decreases health and vigour. *Ojo kshaya* also decreases bala (bala haani), which causes weakening of musculoskeletal functions and immune functions [10], Sootra Sthana/Chapter 17/Verse 73; 17, Sootra Sthana/Chapter 19/Verse 33; 4, Sootra Sthana/Chapter 11/Verse 40]. Second, cognitive decline is associated with low cholesterol in elders [11]. Similarly, *ojo kshaya* is occasionally associated with cognitive distortions (*durmana*), impaired sensorium (*vyathitendreeya*), and obsessive thoughts (*abheekshna dhyana*) [4], Sootra Sthana/Chapter 11/Verse 39–41]. Third, hypocholesterolemia significantly correlates with increased risk of mortality (PQ-9). Similarly, signs of impending death (*arishta lakshana*) are strongly associated with *ojo kshaya*, impaired learning-memory (*smruti kshaya*), and impaired cognition (*satva nasha*) [10], Indreeya Sthana/Chapter 12/Verse 48].

## 2 Conclusions

Walton et al. proposed an equivalence between *ojas* and cholesterol. We add six important lines of evidence to this proposal. First, we propose links between cholesterol and its products at 12 sites of *ojas* activity (PQ5). Second, we link *apara ojas* with circulating cholesteryl esters (LDL-C, HDL-C) and note that both entities respond to diet and drugs (PQs 2, 3). Third, Walton et al. matched products of cholesterol with specific *dhatus*, and we add that cholesterol's 8 products may correlate with *Dhatu tejo* *rupi ojas* [PQ-4]. Notably, other complex lipids [phospholipids, sphingolipids, and glycolipids], cannot perform any of the vital and specialized functions of these 8 products of cholesterol [Fig. 1]. Fourth, we discuss physiological and biochemical mechanisms linking cholesterol and its products with major functions of ojas (*shukra* and *garbhasara*, *bala,* and *vyadhi kshamatva*) (Fig. 1, Fig. 2). Fifth, we explain how hypocholesterolemia and *ojo kshaya* have similar clinical manifestations (PQ-9). Sixth, we propose HDL-C as a marker of healthy *Apara Ojas*. We explain how these 6 points provide correlative and potentially causal evidence for the hypothesis proposing cholesterol and products of cholesterol, as candidate biomarker for *Ojas*. However, this hypothesis needs to be proven by suitable clinical studies.

Our hypothesis has at least 3 clinical applications.

1. Clinicians routinely measure levels of total cholesterol, LDL-C and HDL-C. This data can be better interpreted if status of *ojas* is examined in parallel, by vaidyas. With this approach, a doctor/vaidya could better assess overall vigour, immune competence, and mental health of a patient. The patient can then be advised on strategies to potentially increase/stabilize *ojas.*
2. Most doctors flag hyper-cholesterolemia or borderline hypercholesterolemia and prescribe statins for lowering LDL-C. But, it is equally important to flag hypocholesterolemia [especially in elders], and warn patients of its consequences. In parallel, clinical research should be done to test the potential link between hypocholesterolemia and loss of *Ojas*.
3. Another important question concerns obesity. Why do certain obese individuals maintain health, while others develop metabolic syndrome? There are genetic factors at play, but careful correlative studies and scoring multiple phenotypic parameters [*ojas* status, lipid profile, vagus activity, sleep, stress responses, etc] may add insights. It is a topic that Integrative medicine experts can consider.

## Author contributions

VNS: Conceptualization, validation, formal analysis, investigation, resources, data curation, writing and reviewing original draft, visualization.

PPN: Conceptualization, validation, formal analysis, investigation, resources, data curation, reviewing and editing.

## Declaration of generative AI in scientific writing

Both Authors of this manuscript declare that no form of AI was used in writing and revising this manuscript. No form of AI was used for preparation of the two figures and one table in this manuscript.

## Funding sources

Authors did not receive any grant from funding agencies in the public, commercial, or not-for-profit sectors.

## Conflict of interest

Authors certify that they have no affiliations or involvement in any entity with respect to subject matter discussed in this manuscript.
