# Supplementary figures and images for: Can cholesterol and its products serve as biomarkers for ojas?
Source: J Ayurveda Integr Med. 2025 Nov 24;16(6):101240. doi: 10.1016/j.jaim.2025.101240 (PMC12682015; doi:10.1016/j.jaim.2025.101240)

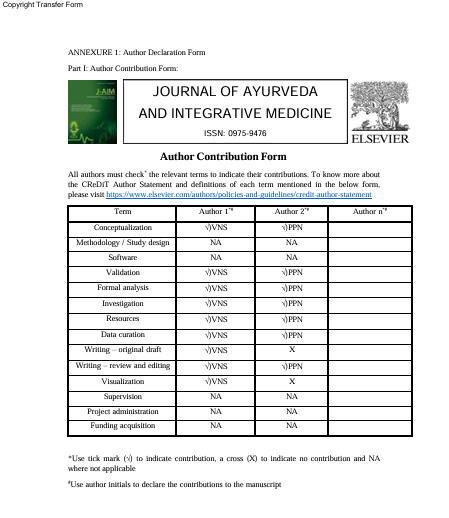


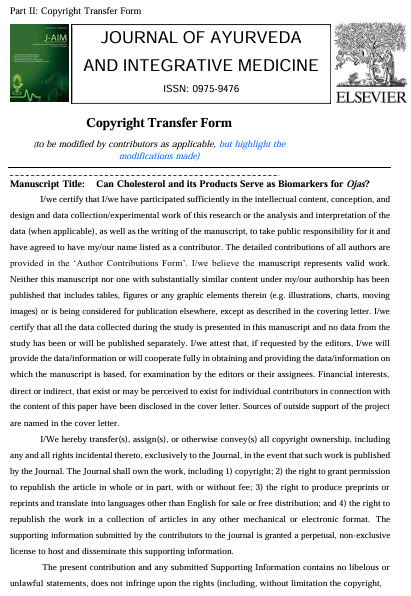


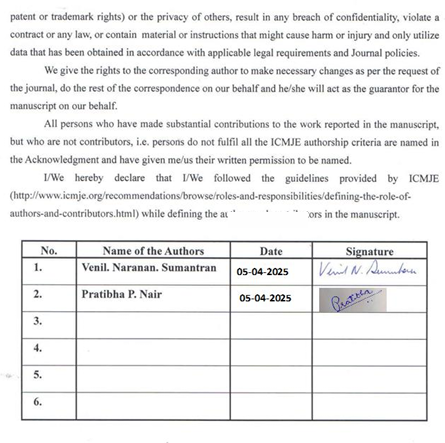

Supplement: Multimedia component 1 [file mmc1.docx]
